# PHD Finger Protein 19 Enhances the Resistance of Ovarian Cancer Cells to Compound Fuling Granule by Protecting Cell Growth, Invasion, Migration, and Stemness

**DOI:** 10.3389/fphar.2020.00150

**Published:** 2020-02-28

**Authors:** Shanming Ruan, Haizhong Zhang, Xinxin Tian, Zhiqian Zhang, Hong Huang, Chao Shi, Wenhong Liu, Xiawei Jiang, Dawei Huang, Fangfang Tao

**Affiliations:** ^1^ Department of Medical Oncology, First Affiliated Hospital of Zhejiang Chinese Medical University, Hangzhou, China; ^2^ Department of Otolaryngology, Head and Neck Surgery, The Second Hospital of Hebei Medical University, Shijiazhuang, China; ^3^ International Joint Academy of Biomedicine, Tianjin, China; ^4^ State Key Laboratory of Medicinal Chemical Biology, Nankai University, Tianjin, China; ^5^ Department of Chinese Medical Formulae, Basic Medical College, Zhejiang Chinese Medical University, Hangzhou, China; ^6^ Department of Immunology and Microbiology, Basic Medical College, Zhejiang Chinese Medical University, Hangzhou, China; ^7^ Department of Chinese Medicine, First Affiliated Hospital of Zhejiang Chinese Medical University, Hangzhou, China

**Keywords:** ovarian cancer, compound fuling granule, PHF19, drug resistance, cancer progression

## Abstract

Ovarian cancer is one of the most common gynecological malignancies in women worldwide with a poor survival rate. We have previously reported that compound fuling granule (CFG), a traditional Chinese medicinal preparation used to treat ovarian cancer in China for over 20 years, significantly promotes cell cycle arrest, apoptosis, senescence, TGFβ-induced invasion and migration, tumor growth, and distant metastasis in ovarian cancer cells. However, the underlying mechanisms are not clear. In the present study, we found that PHF19 expression in ovarian cancer cells positively correlated with their resistance ability to CFG. In addition, PHF19 overexpression increased the resistance of HEY-T30 and SKOV3 cells to CFG, while knockdown of PHF19 enhanced their sensitivity to CFG. Moreover, CFG significantly inhibited the expression of PHF19 both in mRNA and protein levels in these cells. Gain of function and loss of function experiments further proved that PHF19 is a crucial mediator involved in the ovarian cancer progression, including cell proliferation, invasion, migration, and stemness. Importantly, rescue the expression of PHF19 reverted CFG-induced suppression in ovarian cancer cell growth, EMT and stemness, while PHF19 knockdown accelerated CFG’s anti-tumor effect. Overall, our results provide a series of evidence to reveal that PHF19 is critical suppressor for CFG’s anti-tumor effect in ovarian cancer.

## Introduction

Ovarian cancer is considered to be one of the most common gynecological tumors in women worldwide with the highest mortality rate (Kim et al., 2012). Although there are many new advances in strategies for ovarian cancer treatment, the 5-year survival rate is below 45% (Webb and Jordan, 2017). Up to now, the clinical strategy for ovarian cancer is still reduction surgery supplemented by platinum-therapy, while 15%–25% of ovarian cancer patients appeared primary resistant to platinum, and over 80% of patients eventually develop secondary resistance to this drug (Bartels et al., 2019; Pokhriyal et al., 2019; Tsibulak et al., 2019). Therefore, new strategies were urgently needed for improving survival rate and life quality of ovarian cancer patients.

Compound fuling granule (CFG) is a traditional Chinese medicinal preparation widely used in clinical practice for ovarian cancer in China. In clinical experience, CFG can promote blood circulation, enhance chemotherapy efficacy, suppress distant metastasis and improve life quality (Tao et al., 2016). *In vitro* experiments demonstrate that CFG can suppress ovarian cancer cell proliferation and epithelial-to-mesenchymal transition (EMT) (Tao et al., 2016). Meanwhile, *in vivo* animal experiments also reveal that administration of CFG inhibits tumor growth and metastasis to lung (Tao et al., 2016). Moreover, CFG disrupts the mitochondrion-related energy metabolisms in ovarian cancer cells (Ruan et al., 2018). However, the molecular mechanisms underlying CFG’s function remains poorly understood.

PHD finger protein 19 (PHF19), also called PCL3, is an essential component of polycomb repressive complex 2 (PRC2) that functions as a transcriptional repressor in regulating developmental regulatory genes. Human PHF19 gene was first identified in 2004 and its products are markedly overexpressed in many types of cancers, including colon, skin, lung, rectal cervical, uterus, and liver cancers (Wang et al., 2004). Moreover, this increase in expression correlated with cancer progression (Wang et al., 2004). After that, accumulating evidence uncover the oncogenic role of PHF19 in a wide range of tumors (Ghislin et al., 2012; Xu et al., 2015; Lu et al., 2018; Tao et al., 2018b; Ren et al., 2019). For instance, PHF19 knockdown reduces the cell proliferation rate and increases the migration capacities of melanoma cells (Ghislin et al., 2012). In myeloma, PHF19 promotes its tumorigenesis through activating PRC2 complex (Ren et al., 2019). In our previous study, we have also preliminarily proved that PHF19 may act as an oncogene in ovarian cancer SKOV3 cells by using RNAi technology (Tao et al., 2018b), however, its exact role in the biological behaviors of ovarian cancer, especially its role in drug-resistance, needs to be further investigated. Moreover, the findings that CFG exerts an anti-tumor effect and PHF19 functions as an oncogenic role in ovarian cancer promote us to examine the relationship between CFG and PHF19 in ovarian cancer.

In the present study, we found that the resistance ability of ovarian cancer cells to CFG positively correlated with PHF19 expression, which promoted us to determine the response of ovarian cancer cells with PFH19 overexpression or knockdown to CFG. The results demonstrated that overexpression of PHF19 increased cell resistance to CFG and knockdown of PHF19 reduced their resistance to CFG. Additionally, CFG obviously suppressed both the mRNA and protein levels of PHF19 in HEY-T30 and SKOV3 cells. Functional experiments further confirmed that PHF19 was involved in ovarian cell proliferation, invasion, migration and stemness. Moreover, rescue the expression of PHF19 reverted CFG-induced suppression in the cell growth, EMT and stemness of ovarian cancer cells, while knockdown of PHF19 accelerated the anti-tumor effect of CFG. Taken together, our findings provide systematic evidence that PHF19 serves as an important suppressor for the anti-tumor effect of CFG in ovarian cancer.

## Materials and Methods

### Cell Culture

Human HEY-T30 and SKOV3 cells were obtained from the American Type Culture Collection (ATCC; Manassas, VA, USA). HEY-T30 cells and SKOV3 cells were grown in RPMI 1640 medium (Gibco, Grand Island, NY, USA) and McCoy’s 5A medium (Gibco), respectively, supplemented with 10% fetal bovine serum (FBS; Gibco), 100 µg/ml streptomycin (Sigma, St. Louis, MO, USA), and 100 U/ml penicillin (Sigma). All cells were incubated at 37°C in 5% CO_2_.

### CFG Preparation

The CFG was prepared as previously described (Tao et al., 2016). Briefly, four constituent herbs, Radix aconite Lateralis praeparata, Patrinia heterophylla, Poria cocos and Radix paeoniae rubra were combined in the ratio of 1:1:1:1, and extracted with 75% ethanol for (1:10, w/w) twice, 1 h each time (Tao et al., 2016).

### Quantitative RT-PCR (RT-qPCR)

Total RNA was extracted from cells using the TRIzol reagent (Invitrogen, Carlsbad, CA, USA) following the manufacturer’s procedure and then reversely transcribed to cDNA product using the PrimeScript™ 1st Strand cDNA Synthesis Kit (Takara, Dalian, China). Then, RT-qPCR was performed on ABI 7500 Real-Time PCR System using the TB Green^®^ Fast qPCR Mix (Takara). The GAPDH was used as an internal control. The following primers were used: PHF19 forward: ACTCGGGACTCCTATGGTGC, PHF19 reverse: CCTCCGTCAGTTTGGACATCA; GAPDH forward: GGTGGTCTCCTCTGACTTCAACA, GAPDH reverse: GTTGCTGTAGCCAAATTCGTTGT. RT-qPCR for miR-211 detection was performed as previously described (Tao et al., 2018b).

### Western Blot Analysis

Western blot analysis was performed as previously described (Zhu et al., 2019). Primary antibodies were used at a dilution of 1:1000. Horseradish peroxidase (HRP) conjugated goat anti-mouse and anti-rabbit secondary antibodies were used at a dilution of 1:5000. PHF19 antibody (Cat. No. #77271), BCL-2 antibody (Cat. No. #15071), BAD antibody (Cat. No. #9292), E-CADHERIN antibody (Cat. No. #14472), N-CADHERIN antibody (Cat. No. #13116), VIMENTIN antibody (Cat. No. #5741), and β-ACTIN antibody (Cat. No. #3700) were purchased from Cell Signaling Technology (CST; Danvers, MA, USA). OCT4 (Cat. No. ab18976), NANOG (Cat. No. ab109250), SOX2 (Cat. No. ab137385), KLF4 (Cat. No. ab215036), HRP-conjugated goat anti-mouse IgG H&L (Cat. No. ab205719), and anti-rabbit IgG H&L (Cat. No. ab205718) were purchased from Abcam (Cambridge, UK).

### Immunofluorescence Assay

Twenty-four hours or 48 h post treatment of HEY-T30 and SKOV3 with CFG (3 mg/ml or 12 mg/ml), cells were fixed with 4% paraformaldehyde (PFA; Sangon, Shanghai, China) for 15 min and permeabilized by 0.2% Triton X-100 (Sigma) for 5 min at room temperature (RT). Fixed cells were washed twice in TBS and blocked for 1 h with 5% BSA in TBS and then incubated with an anti-PHF19 antibody (CST, Cat. No. #77271) at 4 °C overnight, washed three times in PBS and incubated with goat anti-rabbit IgG H&L (Alexa Fluor^®^ 488; Abcam, Cat. No. #150077) for 1 h at room temperature. Finally, cells were incubated with 10 μg/ml DAPI (Solarbio, Beijing, China) for 10 min at room temperature. Images were captured under a fluorescence microscope (OlymPus BX53; Tokyo, Japan).

### Lentivirus Production

Lentivirus overexpressing PHF19 CDS (Ubi-MCS-3FLAG-SV40-puromycin) and PHF19 shRNAs (hU6-MCS-CMV-Puromycin) were produced by GeneChem (Shanghai, China) as previously described (Tao et al., 2018b). The oligonucleotides synthesized for construction of PHF19 shRNA plasmids were as follows: PHF19 shRNA -1: CCGGCCTCGTGACTTTCGAAGATAACTCGAGTTATCTTCGAAAGTCACGAGGTTTTTG; PHF19 shRNA -2: CCGGCCCACCTCAAGTCATCTATCACTCGAGTGATAGATGACTTGAGGTGGGTTTTTG.

### Cell Proliferation and Colony Formation Assays

For cell proliferation assay, HEY-T30 and SKOV3 were infected with PHF19 CDS or PHF19 shRNA lentivirus for 72 h and a total of 5×10^3^ infected cells were plated into 96-well plates for 24 h. Then, the proliferation was monitored for 8 days. In every 2 days, MTT reagent (Solarbio) was added to each well and incubated for 4 h. Then, culture medium was removed and 100 µl of DMSO was added and the absorbance was measured at 570 nm. Soft agar colony formation assay was performed as previously described (Tao et al., 2018a).

### Apoptosis Detection

Apoptosis assay was performed by an Annexin V-FITC/PI Apoptosis Detection Kit (Solarbio) according to the manufacturer’s manual. Briefly, after harvesting the treated cells, they were washed with PBS three times and resuspended with 100 μl of 1× Annexin V binding buffer. Then, 5 μl of FITC-Annexin V and PI were added to each sample with incubation for 15 min at RT in the dark. Stained cells were washed with 1 ml of 1× Annexin V binding buffer once and resuspended with 400 μl of PBS. Flow cytometry (BD LSRFortessa™; BD Biosciences, Franklin Lakes, NJ, USA) was applied to analyze the population of early and late apoptotic cells.

### Cell Viability Determination

A total of 2×10^4^ wildtype HEY-T30, SKOV3, OVCAR3, and A2780 cells, as well as HEY-T30 or SKOV3 cells infected with PHF19 CDS or PHF19 shRNA lentivirus for 72 h were seeded as triplicate in 96-well plates and then were treated with 0, 0.75, 1.5, 3, 6, 9, 12, 18, and 24 mg/ml of CFG, or treated with 0, 2, 4, 8, 16, 32, 64, 128, 256, and 512 μM of cisplatin (MCE, Shanghai, China), respectively. Twenty-four hours later, 10 μl of 5 mg/ml MTT (Solarbio) was added to the cells for 4 h. Then, the supernatant was discarded and the formazan crystals were dissolved in DMSO (100 μl) for 10 min. At last, the plates were placed on a microplate autoreader (BioTek, Winooski, VT, USA) and the absorbance was detected at 570 nm.

### Transwell Invasion and Migration Assays

**Figure 1:**
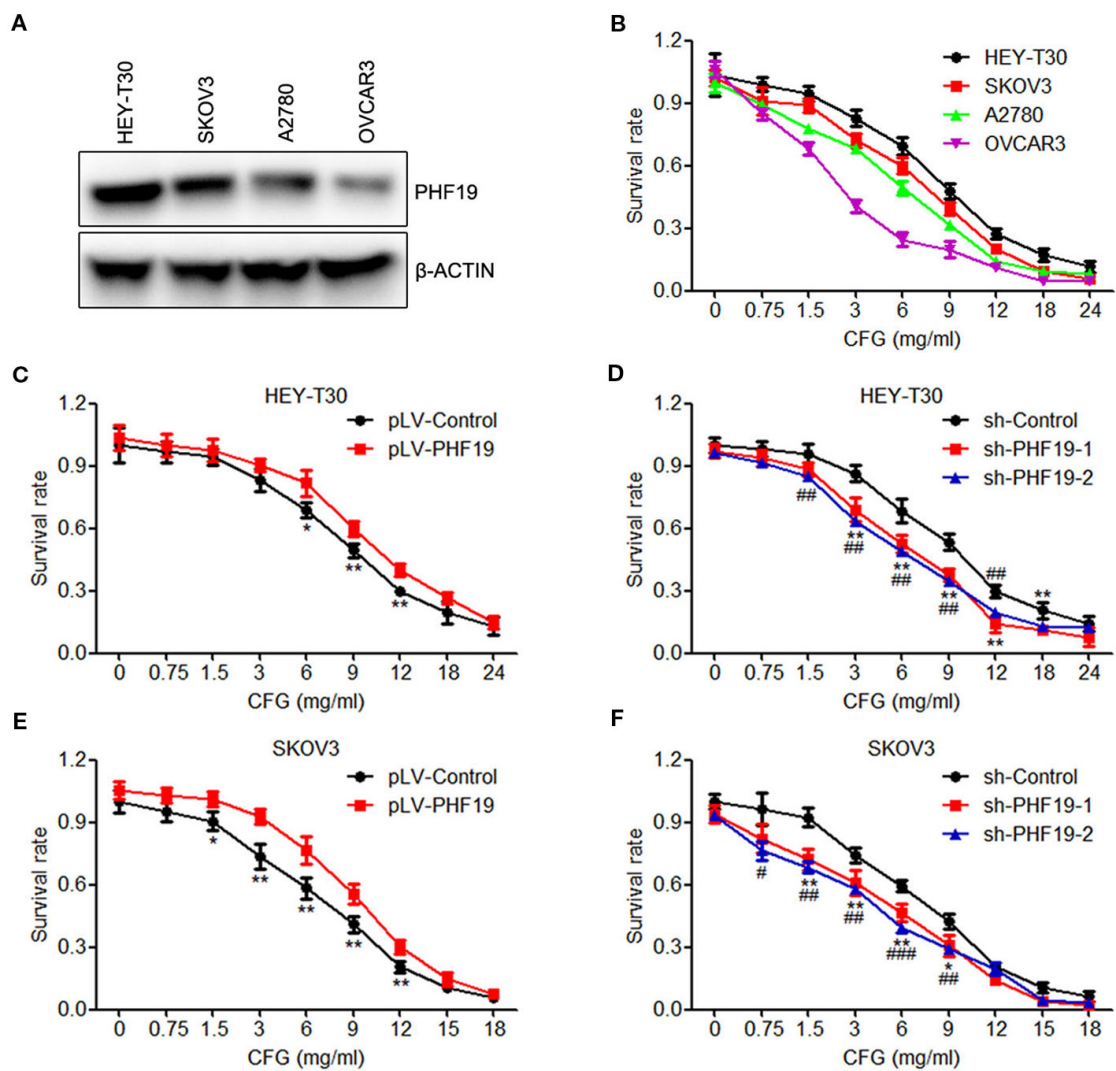
PHF19 enhances the resistance of ovarian cancer HEY-T30 and SKOV3 cells to CFG. **(A)** The protein level of PHF19 in ovarian cancer HEY-T30, SKOV3, A2780 and OVCAR3 cells were determined by Western blot analysis. **(B)** HEY-T30, SKOV3, A2780, and OVCAR3 cells were treated with 0, 0.75, 1.5, 3, 6, 9, 12, 18, and 24 mg/ml of CFG for 24 h and cell viability was detected by MTT assay. **(C, E)** Cell viability was determined in HEY-T30 **(C)** and SKOV3 **(E)** cancer cells treated with different concentrations of CFG in the presence or absence of PHF19 overexpression. The data are shown as average ± SD from three different experiments. ^*^, *P* < 0.05; ^**^, *P* < 0.01. **(D, F)** Different concentrations of CFG were administered to the HEY-T30 **(D)** and SKOV3 **(F)** cancer cells in the presence or absence of PHF19 knockdown and cell viability was assayed. The data are shown as average ± SD from three different experiments. ^*^ and ^#^, *P* < 0.05; ^**^ and ^##^, *P* < 0.01; ^###^, *P* < 0.001; ^*^ and ^**^: sh-Control *v.s.* sh-PHF19-1; ^#^, ^##^ and ^###^: sh-Control *v.s.* sh-PHF19-2.

For migration assay, HEY-T30 and SKOV3 were infected with PHF19 or shPHF19 lentivirus for 72 h and treated with 3 mg/ml of CFG for 24 h. Then, 5×10^4^ (for **Figures 4A, C**) or 1×10^5^ (**Figures 7A, C** and **Figure S2D**) cells were resuspended with 100 μl of fresh basic culture medium and seeded into the top chamber of a Transwell insert (pore size: 8 μm, Corning, NY, USA). The lower chamber was incubated with culture medium with 10% FBS. Twenty-four hours later, the insert was fixed with 4% PFA for 30 min. Non-migrated cells on the top surface of the insert were removed by a cotton swab and migrated cells on the lower surface of the insert were air-dried and stained with 0.05% crystal violet (Sigma) for 30 min. Images of cells on the Transwell membrane were taken with a microscope (Olympus) at 200× magnification and cell numbers of six random fields were counted. For invasion assay, 20 μl of growth factor-reduced Marigel (Corning) were even plated onto the upper chamber of the insert.

### Spheroid Formation Assay

A total of 2×10^3^ cells were seeded into 24-well Corning™ Costar™ Ultra-Low Attachment Microplates and were cultured in serum-free DMEM-F12 (Gibco) supplemented with 1× B27 (Gibco), 20 ng/ml recombinant human EGF (Gibco) and 10 ng/ml recombinant human FGF (Gibco) for 14 days. Images of spheres were taken with a microscope (Olympus). Spheroid numbers and average diameters of six random fields were counted.

### Determination of Cancer Stem Cell Population

ALDH enzymatic activity was assessed using the ALDEFLUOR kit (Stem Cell Technologies, Vancouver, BC, Canada) following the manufacturer’s protocol. Cells treated with ALDH inhibitor DEAB were used as a negative control. The surface markers of ovarian cancer stem cells CD44 (FITC anti-human CD44; Cat. No. 338804; Biolegend, San Diego, CA, USA) and CD117 (APC anti-human CD117; Cat. No. 313206; Biolegend) were also stained for determination of cancer stem cells. Flow cytometry (BD LSRFortessa™) was applied to analyze the ALDH^+^ and CD44^+^CD117^+^ cells.

### Statistical Analysis

All analysis was performed by SPSS version 17.0. Statistical significance was determined using student’s *t*-test or one-way ANOVA analysis. Data were presented as mean ± SD. P-value < 0.05 was considered as statistically significant.

## Results

### PHF19 Protects the Resistance of HEY-T30 and SKOV3 Cells to CFG

Initially, to determine whether PHF19 is involved in the regulation of drug-resistance of ovarian cancer cells to CFG, we detected the protein expression of PHF19 in four ovarian cancer cell lines HEY-T30, SKOV3, A2780, and OVCAR3 and determined the viability of these cell lines with different doses of CFG treatment. The results showed that the resistance ability of these cells to CFG positively correlated with PHF19 expression (**Figures 1A, B**). Then, the viability of HEY-T30 and SKOV3 ovarian cancer cells with/without PHF19 overexpression or knockdown was evaluated in the presence of different concentrations of CFG (HEY-T30: 0, 0.75, 1.5, 3, 6, 9, 12, 18, and 24 mg/ml; SKOV3: 0, 0.75, 1.5, 3, 6, 9, 12, 15, and 18 mg/ml) for 24 h. As shown in **Figures 1C, E**, the viability of HEY-T30 and SKOV3 cells showed a gradual decrease with increasing concentrations of CFG. PHF19 overexpression increased the resistance of these cells to CFG (**Figures 1C, E**). On the contrary, knockdown of PHF19 in HEY-T30 or SKOV3 cells further decreased their viability after CFG treatment (**Figures 1D, F**). On the other hand, we also examined the effect of PHF19 on ovarian cancer cells with cisplatin treatment. We found that overexpression or knockdown of PHF19 could not influence the chemoresistance of HEY-T30 and SKOV3 to cisplatin (**Figure S1**), suggesting that the drug-resistance of ovarian cancer cells induced by PHF19 may be specific for CFG.

### CFG Suppresses the mRNA and Protein Levels of PHF19 in Dose- and Time- Dependent Manners

**Figure 2:**
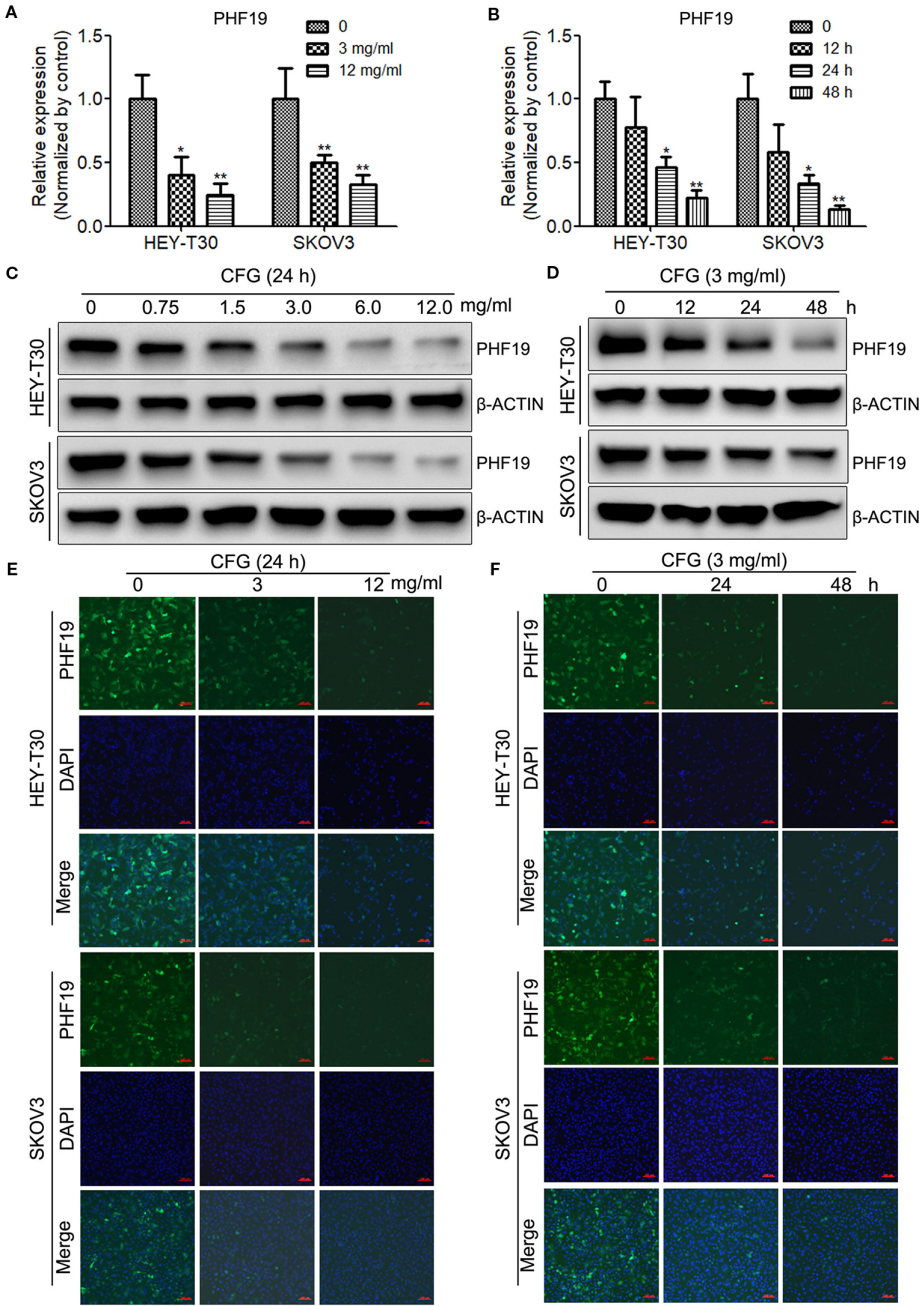
CFG suppresses the mRNA and protein levels of PHF19 in time- and dose- dependent manners. **(A)** HEY-T30 and SKOV3 cells were treated with 0, 3 or 12 mg/ml of CFG for 24 h, and subjected to RT-qPCR to detect the mRNA expression of PHF19. **(B)** RT-qPCR were performed to determine the mRNA expression of PHF19 in HEY-T30 and SKOV3 cells treated with 3 mg/ml of CFG for 0, 12, 24, or 48 h, respectively. **(C)** Western blot analysis to examine the protein expression of PHF19 in HEY-T30 and SKOV3 cells treated with 0, 0.75, 1.5, 3, 6, or 12 mg/ml of CFG for 24 h. **(D)** HEY-T30 and SKOV3 cells were treated with 3 mg/ml of CFG for 0, 12, 24, or 48 h, respectively. Then, Western blot analysis to examine the protein expression of PHF19. **(E, F)** Immunofluorescence examination of PHF19 expression in HEY-T30 and SKOV3 cells treated with CFG at a concentration of 0, 3 or 12 mg/ml for 24 h **(E)** or at a dose of 3mg/ml for 0, 24, or 48 h. (green, PHF19; blue, DAPI; scale bars = 100 μm). Data are the mean ± SD of three independent experiments. ^*^, *P* < 0.05; ^**^, *P* < 0.01.

Next, to gain insight into whether CFG influences the expression of PHF19, HEY-T30, and SKOV3 cells were treated with different concentrations of CFG (0, 3 and 12 mg/ml) for 24 h. RT-qPCR results showed that CFG inhibited the mRNA level of PHF19 in HEY-T30 and SKOV3 cells in a concentration-dependent manner (**Figure 2A**). Next, we performed time-course experiments in the presence of 3 mg/ml of CFG and quantitated the mRNA expression of PHF19 at time points 0, 12, 24, and 48 h. The results also indicated that gradually decreased PHF19 expression occurred from 12 to 48 hours after CFG treatment (**Figure 2B**). Western blot analysis (**Figures 2C, D**) and immunofluorescence assay (**Figures 2E, F**) also proved that CFG suppressed the protein level of PHF19 in dose- and time- dependent manners.

### PHF19 Promotes Cell Proliferation and Inhibits Apoptosis in Ovarian Cancer HEY-T30 and SKOV3 Cells

**Figure 3:**
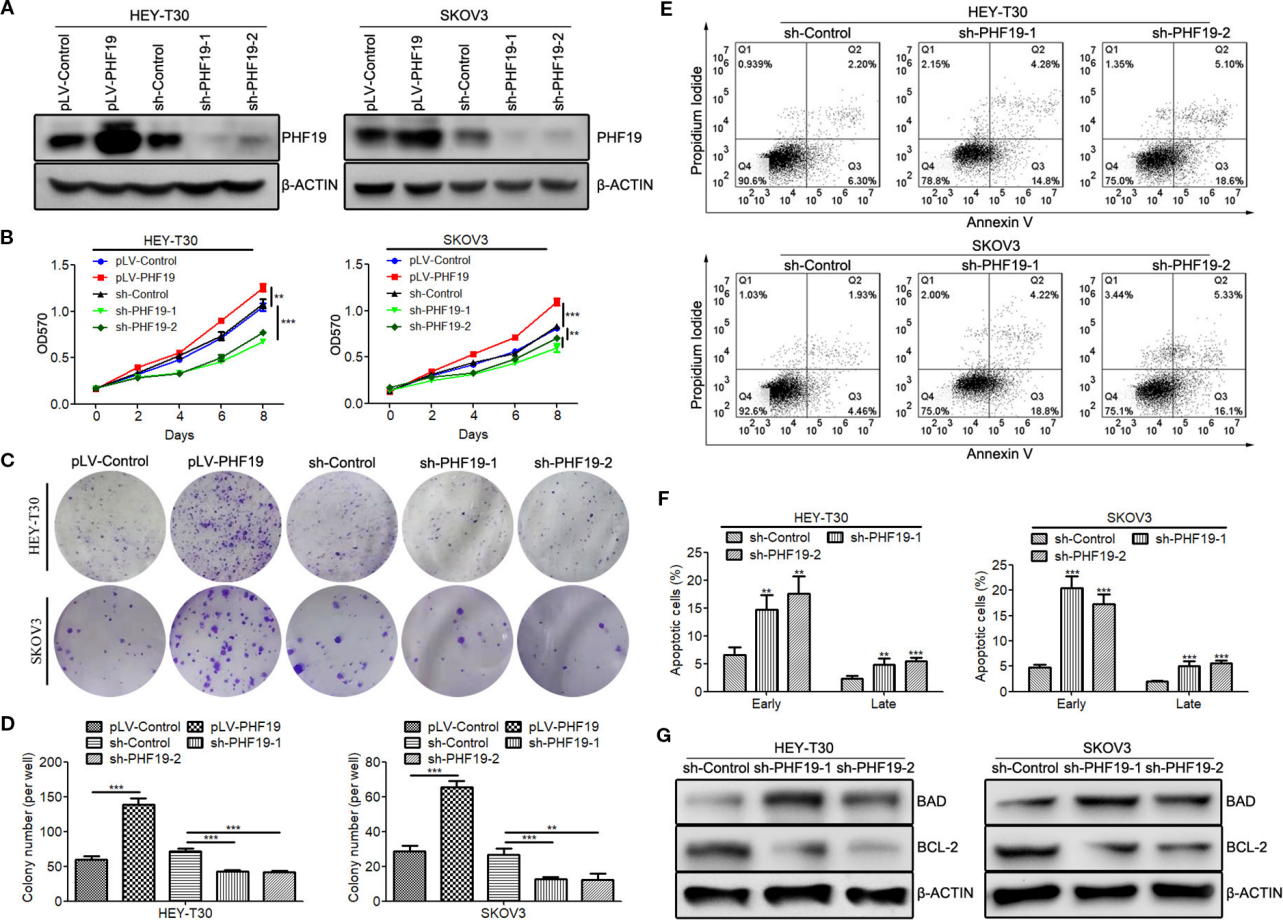
PHF19 promotes proliferation and suppresses apoptosis in ovarian cancer HEY-T30 and SKOV3 cells. **(A)** After overexpression of PHF19 or knockdown of PHF19 by infecting with corresponding lentivirus in HEY-T30 and SKOV3 cells, PHF19 protein expression was determined by Western blot analysis. **(B)** Cell proliferation was evaluated by MTT assay. After infection of PHF19 overexpression lentivirus or PHF19 shRNA lentivirus, OD_570_ values were detected. ANOVA was analyzed to compare cell proliferation curves. **(C, D)** Colony formation assay to examine the clonogenic ability of HEY-T30 and SKOV3 cells infected with PHF19 overexpression lentivirus or PHF19 shRNA lentivirus. Representative pictures are shown in **(C)** and quantitative analysis of colony numbers is shown in **(D)**. **(E, F)** Cell apoptosis rates were detected in HEY-T30 and SKOV3 cells with PHF19 knockdown using flow cytometry. Representative images are shown in **(E)** and early and late apoptotic cells were analyzed in **(F)**. **(G)** BAD and BCL-2 protein expression in HEY-T30 and SKOV3 cells with PHF19 knockdown were determined by Western blot analysis. The data are shown as average ± SD from three different experiments. ^**^, *P* < 0.01; ^***^, *P* < 0.001.

We have previously found that silence of PHF19 suppresses cellular proliferation, migration, and xenograft growth and promotes programmed cell death in SKOV3 cells. In this study, before elucidating the mechanisms of PHF19 underlying the resistance of ovarian cancer cells to CFG, we systematically determine the function of PHF19 in the pathogenesis of ovarian cancer by conducting gain-of-function and lose-of-function experiments. We constructed an overexpression lentivirus (Ubi-MCS-3FLAG-SV40-puromycin-PHF19, also named pLV-PHF19) to upregulate the expression of PHF19 and two shRNA lentivirus (hU6-MCS-CMV-Puromycin-PHF19-shRNA 1 & 2 also named sh-PHF19-1 and sh-PHF19-2) to knockdown the expression of PHF19 in HEY-T30 and SKOV3 cells. After infection with the PHF19 overexpression or shRNA lentivirus for 72 h, we ensured the overexpression and silencing efficiencies using Western blot analysis and all the lentivirus were well functioned (**Figure 3A**). Next, we evaluated the effects of PHF19 overexpression or knockdown on cell proliferation in HEY-T30 and SKOV3 cells using MTT and colony formation assays. As shown in **Figures 3B–D**, upregulation of PHF19 significantly promoted the proliferation rate and colony formation ability of HEY-T30 and SKOV3 cells, whereas downregulation of PHF19 inhibited cell proliferation and colony formation. To evaluate the effect of PHF19 on HEY-T30 and SKOV3 cell apoptosis, apoptotic population was detected by flow cytometry. Compared with the control group, both the early and late apoptotic populations in PHF19-silenced HEY-T30 and SKOV3 cells were significantly increased (**Figures 3E, F**). We also detected the expression of apoptosis-associated proteins BAD and BCL-2 in HEY-T30 and SKOV3 cells infected with PHF19 shRNA or control shRNA lentivirus. We found the expression of pro-apoptotic gene BAD was increased, while the expression of anti-apoptotic gene BCL-2 was decreased in PHF19-silenced cells compared with that of control cells (**Figure 3G**). These results confirmed that PHF19 can function as a pro-proliferation regulator in ovarian cancer.

### PHF19 Facilitates Ovarian Cancer HEY-T30 and SKOV3 Cell Invasion and Migration

**Figure 4:**
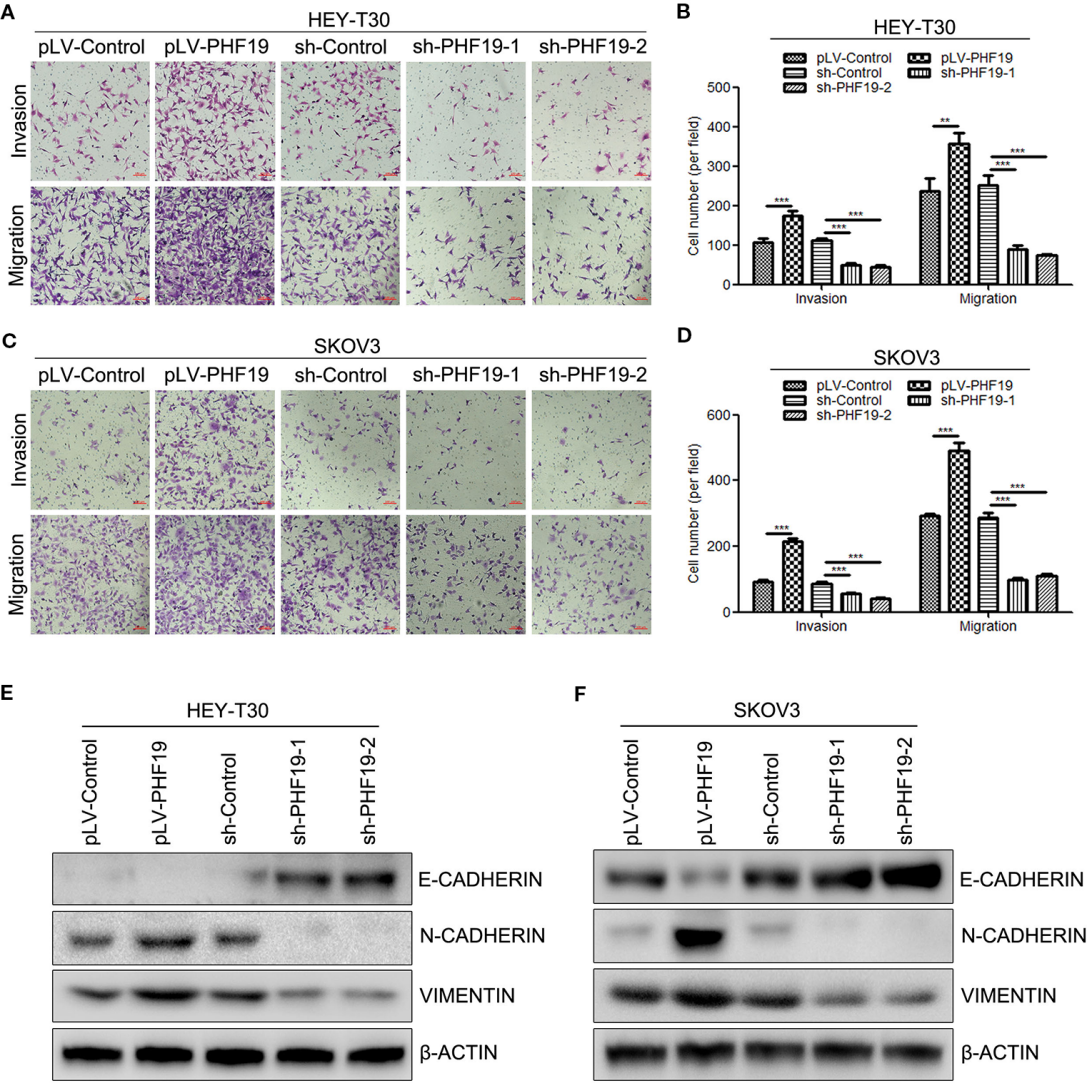
PHF19 promotes invasion and migration in ovarian cancer HEY-T30 and SKOV3 cells. **(A–D)** After overexpression of PHF19 or knockdown of PHF19 by infecting with corresponding lentivirus, invasion and migration abilities in HEY-T30 and SKOV3 cells were detected by Transwell assays. **(A**, **C)** Representative image of cell migration and invasion. Scale bar: 100 μm. **(B**, **D)** Quantitative results of migration and invasion assays. The data are shown as average ± SD from three different experiments. ^**^, *P* < 0.01; ^***^, *P* < 0.001. **(E, F)** Expression of EMT markers, E-CADHERIN, N-CADHERIN, and VIMENTIN was determined by Western blot analysis.

In order to further confirm the role of PHF19 in ovarian cancer cell progression, the cell invasion and migration capacities were determined in HEY-T30 and SKOV3 cells with/without PHF19 overexpression or knockdown by Transwell assays. The invaded and migrated cell numbers of HEY-T30 and SKOV3 cells with PHF19 overexpression were significantly increased compared with that of the control groups (**Figures 4A–D**). By contrast, PHF19-silenced HEY-T30 and SKOV3 cells showed significantly lower invasion and migration abilities than that of control shRNA-infected cells (**Figures 4A–D**). Progression of cancer cell invasion and migration was usually companied with changes of epithelial-mesenchymal transition (EMT) markers. In this part, we also detected the protein expression of epithelial marker E-CADHERIN and mesenchymal markers N-CADHERIN and VIMENTIN in different groups by Western blot analysis. The results demonstrated that overexpression of PHF19 decreased E-CADHERIN and increased N-CADHERIN and VIMENTIN expression (**Figures 4E, F**), while silence of PHF19 enhanced the protein expression of E-CADHERIN and reduced the protein levels of N-CADHERIN and VIMENTIN (**Figures 4E, F**). These data indicates that PHF19 promotes aggressive cancer phenotypes of ovarian cancer cells *in vitro*.

### PHF19 Increases the Stemness of Ovarian Cancer HEY-T30 and SKOV3 Cells

**Figure 5:**
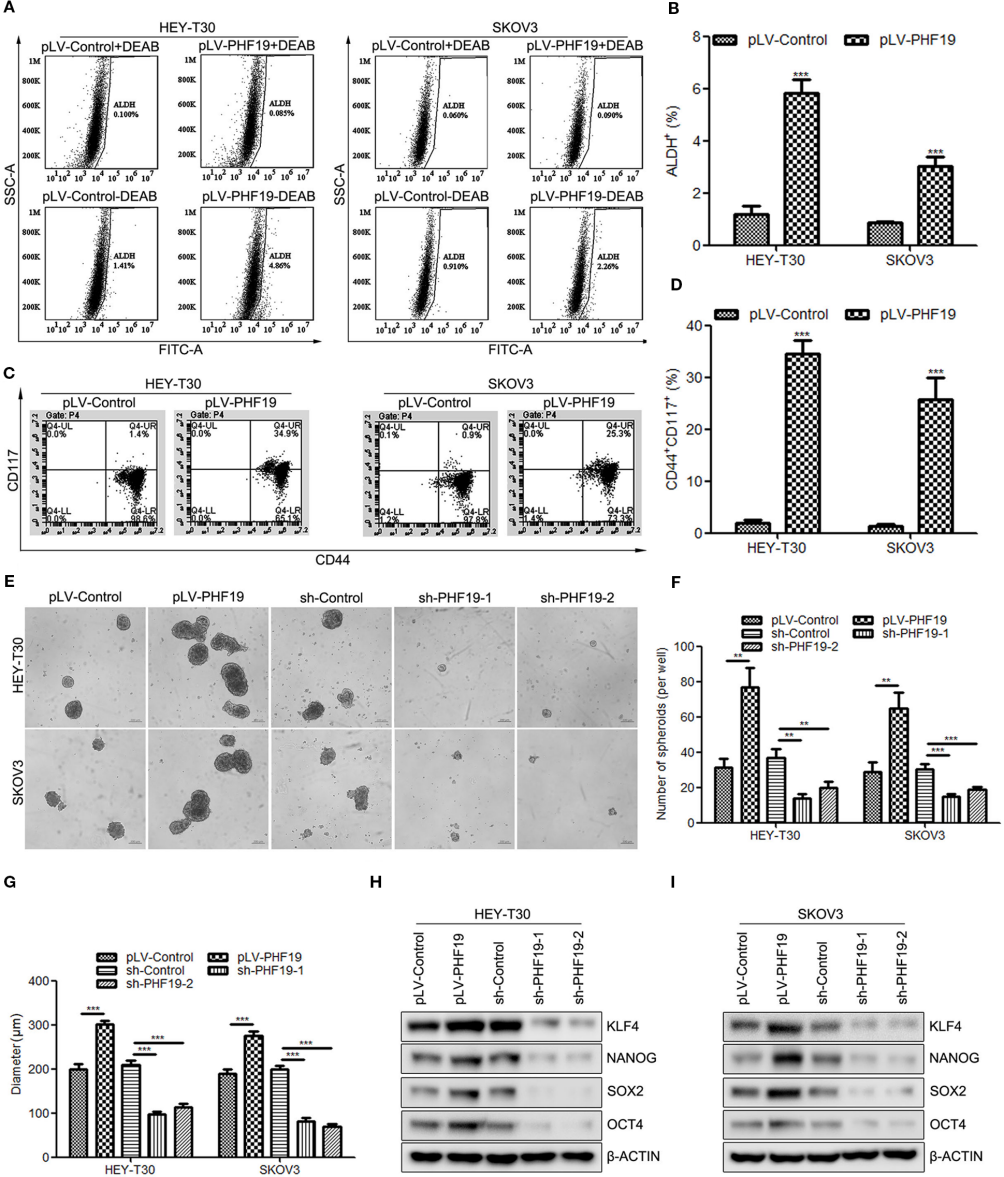
PHF19 enhances tumor stemness in ovarian cancer HEY-T30 and SKOV3 cells. **(A, B)** ALDEFLUOR staining of ALDH^+^ cells in HEY-T30 and SKOV3 infected with PHF19 overexpression lentivirus. **(A)** representative FACS analysis. **(B)** percentage of ALDH^+^ cells. **(C, D)** Flow cytometric analysis of surface markers of CD44 and CD117 in PHF19-overexpressed HEY-T30 and SKOV3 cells. **(C)** representative FACS analysis. **(D)** percentage of CD44^+^CD117^+^ cells. **(E–G)** HEY-T30 and SKOV3 cells with PHF19 overexpression or knockdown were subjected to a sphere formation assay. Scale bar: 200 μm. The number and size of tumor spheres were shown in **(F**, **G)** respectively. **(H, I)** Western blotting analyses were used to determine the expression levels of stem cell markers, KLF4, NANOG, SOX2, and OCT4. The data are shown as average ± SD from three different experiments. ^**^, *P* < 0.01; ^***^, *P* < 0.001.

It is hypothesized that cancer stem cells (CSC) might be the origin of cancers and responsible for the proliferation, EMT, metastasis, recurrence, and chemoresistance of tumor cells (Carnero et al., 2016; Matsui, 2016; Toledo-Guzman et al., 2018). Herein, we sought to determine whether PHF19 plays a role in ovarian cancer stemness. Well-studied ovarian cancer stem cell biomarkers include aldehyde dehydrogenase (ALDH) and CD44/CD117. Firstly, to detect the ALDH positive (ALDH^+^) cells in different groups, HEY-T30 and SKOV3 cells infected PHF19 overexpression lentivirus or control lentivirus underwent ALDEFLUOR assays using flow cytometry. The inhibitor of ALDH enzyme DEAB was used as a negative control. The results demonstrated that PHF19 overexpression significantly increased the population of ALDH^+^ cells both in HEY-T30 and SKOV3 cells (**Figures 5A, B**). We also determined the stem cell population with CD44 and CD117 double staining. The data also showed that ectopic expression of PHF19 sharply elevated the CD44^+^CD117^+^ cell population (**Figures 5C, D**). In addition to the marker analyses, we also examined the effect of PHF19 expression changes on the self-renewal ability of cancer stem cells using sphere formation assay. HEY-T30 and SKOV3 cells with PHF19 overexpression formed larger and more spheres compared to control cells, while silence of PHF19 suppressed the size and number of spheres (**Figures 5E–G**). Western blot analysis also revealed that PHF19 induced the protein expression of stem cell markers KLF4, NANOG, SOX2, and OCT4 and PHF19 knockdown suppressed their expression (**Figures 5H, I**).

### Rescue the Expression of PHF19 Attenuates CFG-Induced Ovarian Cancer Cell Apoptosis

**Figure 6:**
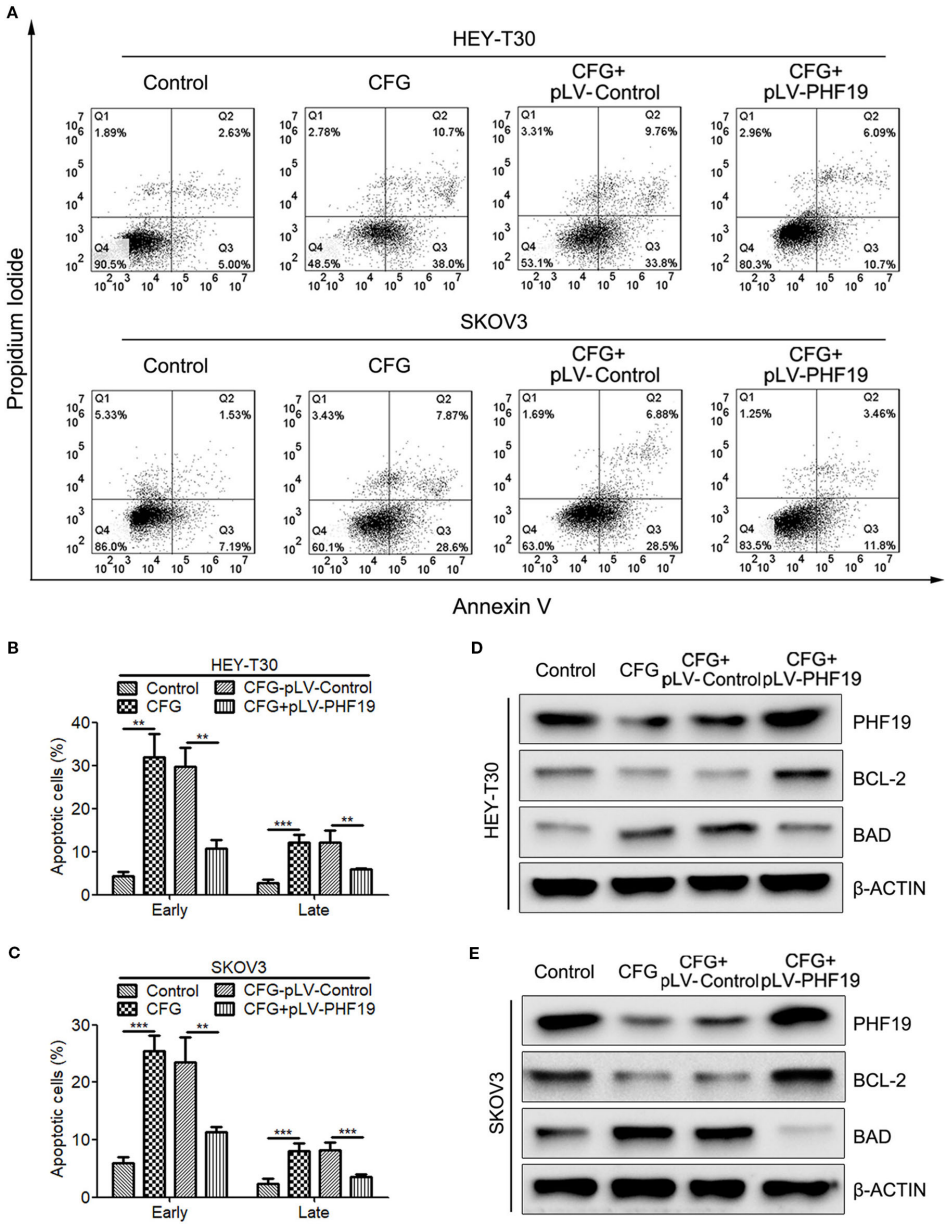
Ectopic expression of PHF19 reverses the promoting effect of CFG on ovarian cancer cell apoptosis. **(A–C)** Apoptosis assay for control and 3 mg/ml of CFG treated cells with/without PHF19 overexpression. The analyses of early and late apoptotic cells were shown in **(B**, **C)**, respectively. The data are shown as average ± SD from three different experiments. ^**^, *P* < 0.01; ^***^, *P* < 0.001. **(D, E)** Western blot analysis of PHF19, BCL-2, and BAD protein expression in control and 3 mg/ml of CFG treated cells with or without PHF19 overexpression.

To further understand the reason why PHF19 enhances the resistance of ovarian cancer cells to CFG, we attempted to performed the following rescue experiments. Apoptosis assay by Annexin V/PI double staining showed that CFG significantly promoted the apoptotic rate of HEY-T30 and SKOV3 cells. Overexpression of PHF19 resulted in decreased apoptotic population of HEY-T30 and SKOV3 cells induced by CFG (**Figures 6A–C**). In addition, CFG treatment suppressed the protein expression of anti-apoptotic gene BCL2 and induced the protein level of pro-apoptotic gene BAD (**Figures 6D, E**). Rescue expression of PHF19 reverted the expression of BCL2 and BAD (**Figures 6D, E**).

### Ectopic Expression of PHF19 Rescues Invasion and Migration Abilities of CFG-Treated Ovarian Cancer Cells

**Figure 7:**
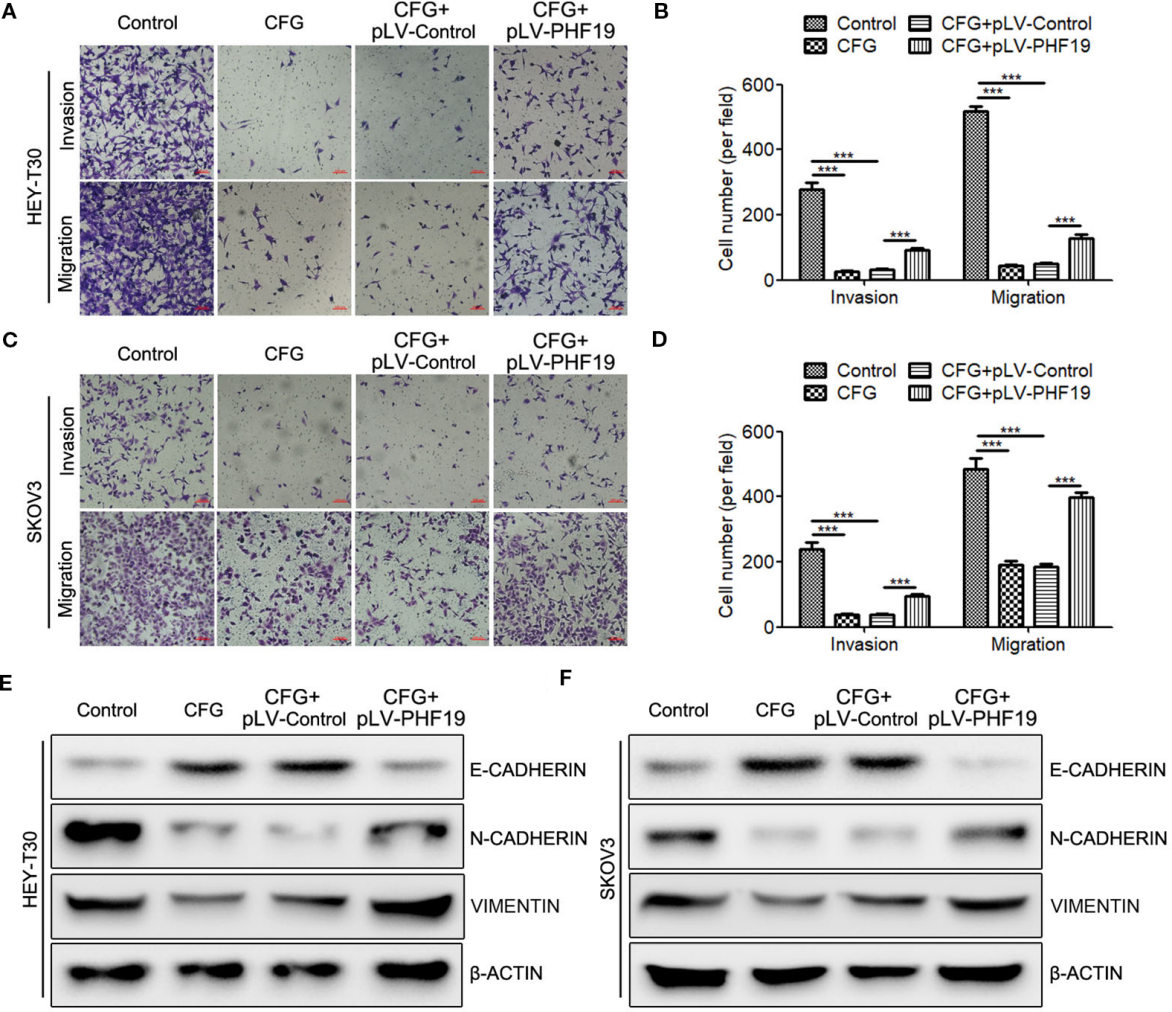
Overexpression of PHF19 attenuates the suppressive effect of CFG on ovarian cancer cell invasion and migration. **(A**–**D)** Transwell assays to determine the invasion and migration capacities of HEY-T30 **(A)** and SKOV3 **(C)** cancer cells treated with 3 mg/ml of CFG in the presence and absence of PHF19 overexpression. **(A, C)** Representative images of cell migration and invasion. Scale bar: 100 μm. **(B**, **D)** Quantitative results of migration and invasion assays. The data are shown as average ± SD from three different experiments. ^***^, *P* < 0.001. **(E**, **F)** Western blot analysis of EMT markers, E-CADHERIN, N-CADHERIN, and VIMENTIN protein expression in control and 3 mg/ml of CFG treated cells with/without PHF19 overexpression.

To further clarify how PHF19 influences CFG’s anti-tumor effect in ovarian cancer. We rescued the expression of PHF19 in CFG-treated HEY-T30 and SKOV3 cells and determined the invasion and migration abilities using Transwell assays. As expected, CFG obviously suppressed ovarian cancer cell invasion and migration compared with the control group (**Figures 7A–D**). Importantly, ectopic expression of PHF19 in CFG-treated HEY-T30 and SKOV3 cells partially attenuated the anti-invasion and anti-migration effects of CFG (**Figures 7A–D**). Moreover, overexpression of PHF19 reversed the induction of epithelial marker E-CADHERIN and attenuated the suppression of mesenchymal markers N-CADHERIN and VIMENTIN caused by CFG (**Figures 7E, F**).

### Rescue of PHF19 Expression Attenuates the Inhibitory Effect of CFG on Ovarian Cancer Cell Stemness

**Figure 8:**
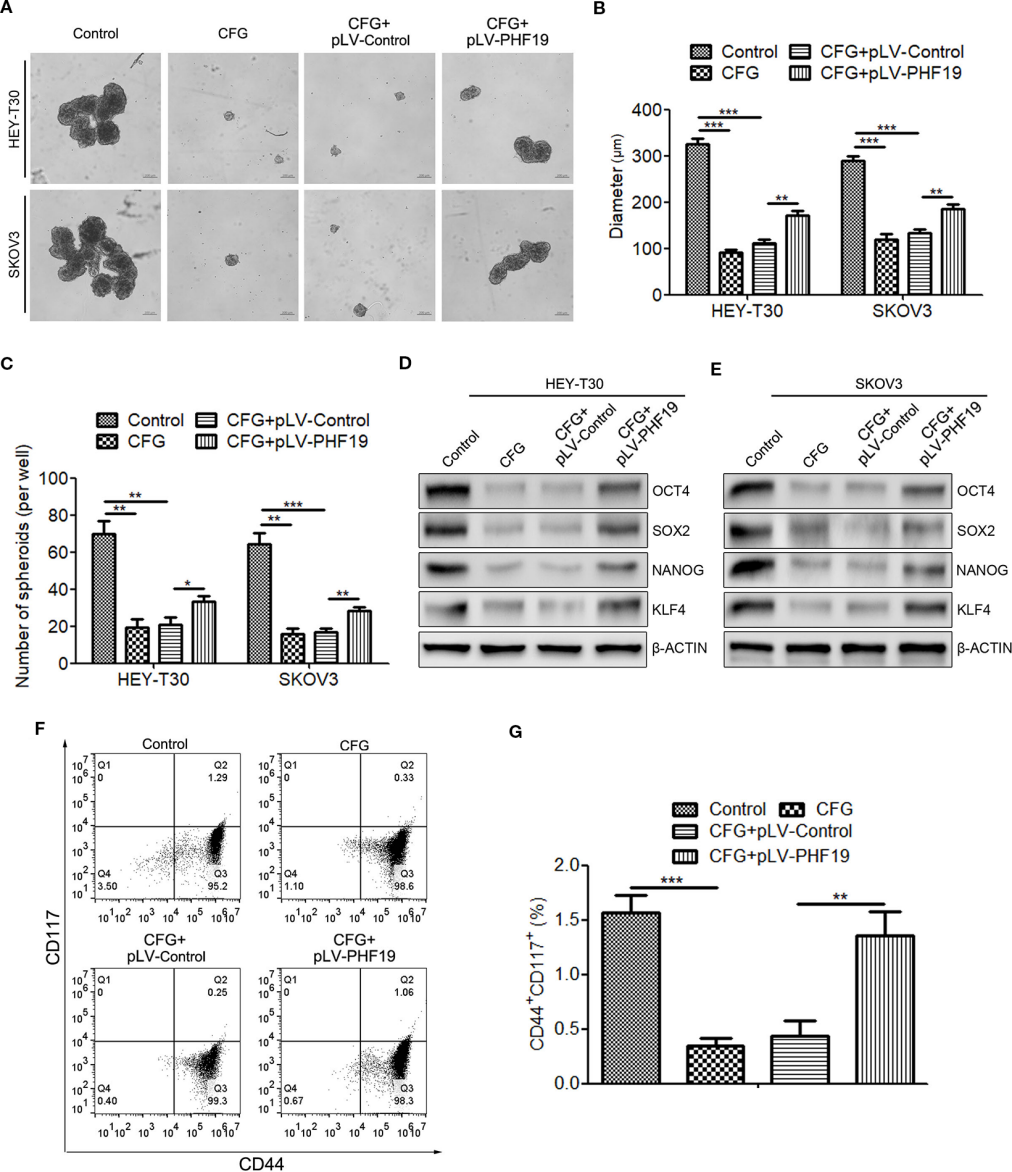
Rescue expression of PHF19 reverts the suppression of tumor stemness ability of HEY-T30 and SKOV3 cells by CFG. **(A**–**C)** Comparison of sphere formation in HEY-T30 and SKOV3 cells treated with 3 mg/ml of CFG in the presence and absence of PHF19 overexpression. **(A)** Representative images. Scale bar: 200 μm. **(B, C)** Quantitative analyses of sphere size **(B)** and number **(C)**. **(D**, **E)** The expression of the stemness associated genes, OCT4, SOX2, NANOG, and KLF4, were determined in control and 3 mg/ml of CFG treated cells with/without PHF19 overexpression by Western blot analysis. **(F**, **G)** Flow cytometric analysis of surface markers of CD44 and CD117 in control and 3 mg/ml of CFG treated cells with/without PHF19 overexpression. The data are shown as average ± SD from three different experiments. ^*^, *P* < 0.05; ^**^, *P* < 0.01; ^***^, *P* < 0.001.

To investigate whether dysregulation of PHF19 expression contributes to the suppression of ovarian cancer cell stemness caused by CFG, sphere formation assay was performed. The results showed that CFG treatment led to the decrease of both size and number of spheres in HEY-T30 and SKOV3 cells (**Figures 8A–C**), while ectopic expression of PHF19 re-elevated the sphere formation capacity (**Figures 8A–C**). In addition, the protein expression levels of stem cell markers OCT4, SOX2, NANOG, and KLF4 were downregulated by CFG, whereas rescue expression of PHF19 reelevated their expression (**Figures 8D, E**). Moreover, CD44 and CD117 double staining also demonstrated that CFG treatment reduced the population of CD44^+^CD117^+^ cells, which could be reverted by overexpression of PHF19 (**Figures 8F, G**).

### Knockdown of PHF19 Accelerates the Anti-Tumor Effect of CFG in Ovarian Cancer

Next, we further evaluated the effect of CFG on ovarian cancer SKOV3 cells with PHF19 knockdown, including cell apoptosis, invasion, migration and stemness. As expected, PHF19 knockdown could accelerate CFG-induced cell apoptosis in ovarian cancer (**Figures S2A–C**). Meanwhile, silence of PHF19 also enhanced the suppression of cell migration (**Figures S2D–F**) and stemness (**Figures S2G–I**) induced by CFG.

Therefore, it can be concluded that PHF19 antagonizes CFG’s anti-tumor effect in ovarian cancer by protecting cell proliferation, invasion, migration, and stemness.

## Discussion

As a cellularly and molecularly heterogeneous disease, ovarian cancer is one of the deadliest gynecological malignancies in women owing to enduring challenges for diagnosis, increasing rate of recurrence, high incidence of metastasis, and developing resistance to chemotherapy (Islam and Aboussekhra, 2019). This study is the first to report the role of PHF19 in the anti-ovarian tumor effect of CFG. CFG is a traditional Chinese medicine (TCM), which has been widely used in the treatment of ovarian cancer for over 20 years in China. CFG consists of four ingredients, Aconitum napellus, Poria cocos F.A. Wolf, Patrinia heterophylla DC, and Radix paeoniae Rubra. Surprisingly, not like the compound, none of these ingredients could reduce the expression of PHF19 alone, suggesting that the interaction among these ingredients and the integrity of this compound are vital for the anti-tumor function of CFG. However, the molecular mechanism by which CFG modulates the expression of PHF19 remains unclear. We have previously found that miR-211 can suppress the expression of PHF19 in ovarian cancer cells by targeting to its 3’ untranslated region (3’ UTR) (Tao et al., 2018b). Here, we also determined the expression of miR-211 in ovarian cancer cells with 0, 3 and 12 mg/ml of CFG treatment for 24 h and found that CFG could not influence the expression of miR-211, suggesting that CFG-induced PHF19 downregulation is not mediated by miR-211 (**Figure S3**). Moreover, interestingly, the protective role of PHF19 in drug-resistance could not be found in cells treated with cisplatin. Nevertheless, more drug should be detected to determine whether the function of PHF19 in CFG’s anti-tumor effect is specific.

During the past several decades, little has been known about the molecular events involved in ovarian cancer. In the present study, we systematically elucidated the role of PHF19 in ovarian cancer proliferation, apoptosis, invasion, migration and stemness, which may help us to find a potential molecular target for the prevention and treatment of ovarian cancer.

PHD finger protein 19 (PHF19) contains a Tudor domain, two PHD finger domains (Lu and Wang, 2013). As a member of polycomblike proteins, it can modulate the recruitment and catalytic activity of polycomb repressive complex 2 (PRC2) (van Mierlo et al., 2019). PRC2 is a unique protein complex with multiple subunits and involved in epigenetic regulation during the development of multicellular organisms. Generally, recruitment of PRC2 to DNA regions of target genes to catalyze the methylation of lysine 27 on histone 3 (H3K27me3), are essential for controlling the developmental gene expression and maintaining cell specification (Kouznetsova et al., 2019). Aberrant expression of PHF19 has been implicated in regulating the pathogenesis and progression of a wide range of cancers, including melanoma (Ghislin et al., 2012), hepatocellular carcinoma (Xu et al., 2015; Cai et al., 2018), glioma (Lu et al., 2018), glioblastoma (Deng et al., 2018), multiple myeloma (Ren et al., 2019). In our previous publication, we have found that patients with higher expression of PHF19 are associated with shorter progression-free survival (PFS) than that with lower PHF19 expression. Functionally, PHF19 knockdown suppresses proliferation, migration, and xenograft growth in SKOV3 cells (Tao et al., 2018b). Moreover, PHF19 is found to be a direct target of miR-211 in ovarian cancer (Tao et al., 2018b). In this study, we further elucidate the exact role of PHF19 in ovarian cancer proliferation, apoptosis, invasion, and migration by a series of gain-of-function and loss-of-function experiments both in HEY-T30 and SKOV3 cells. More importantly, the present study is the first to report that PHF19 enhances tumor stemness in ovarian cancers. Actually, in embryonic stem cells, PHF19 can recruit PRC2 to stem cell genes marked by H3K36me3 and promote silencing of differentiation-associated genes during development *via* the Tudor domain of PHF19 (Ballare et al., 2012; Cai et al., 2013). Here, we also demonstrate that PHF19 can activate the expression of stem cell markers OCT4, NANOG, SOX2, and KLF4, nevertheless, the underlying mechanisms need to be further investigated.

On the other hand, PHF19 encodes two distinct isoforms: a full-length isoform (580 amino acids; also named hPCL3l) with one Tudor domain, two PHD finger domains and a conserved C-terminal chromo-like domain and a shorter isoform (207 amino acids; also called hPCL3s) with only a TUDOR domain and the first PHD domain. In this research, we cloned the longer isoform for PHF19 overexpression. The expression profiles and function of the shorter isoform of PHF19 in ovarian cancer are still unknown.

In conclusion, results of the current study demonstrate that CFG exerts its anti-tumor function in ovarian cancer, at least partially, depending on the downregulation of PHF19.

## Data Availability Statement

All datasets generated for this study are included in the article.

## Author Contributions

FT, DH and ZZ designed the study, wrote, revised and finalized the paper. SR, HZ, XT and HH carried out experiments. SR, XT, ZZ, HH, CS, WL, XJ and DH analyzed experimental results.

## Funding

This research was supported by Zhejiang Provincial Program for the Cultivation of High-Level Innovative Health Talents (No. 2015-43), China Postdoctoral Science Foundation (No. 2015M581292 and 2019T120533), Zhejiang Provincial Programme of TCM (No. 2019ZQ013) and Zhejiang Chinese Medical University Programme (No. 2018ZG23).

## Conflict of Interest

The authors declare that the research was conducted in the absence of any commercial or financial relationships that could be construed as a potential conflict of interest.

## Supplementary Material

Figure S1 Changes of PHF19 expression cannot influence the anti-tumor effect of cisplatin in ovarian cancer HEY-T30 and SKOV3 cells. **(A** and **C)** HEY-T30 **(A)** and SKOV3 **(C)** cells with/without PHF19 overexpression were treated with different concentrations of cisplatin (0, 2, 4, 8, 16, 32, 64, 128 or 256 μM) and cell viability was detected by MTT assays. **(B** and **D)** Different concentrations of cisplatin (0, 2, 4, 8, 16, 32, 64, 128 or 256 μM) were added into the HEY-T30 **(B)** and SKOV3 **(D)** cancer cells with/without PHF19 knockdown and cell viability was assayed. The data are shown as average ± SD from three different experiments.

Figure S2 PHF19 knockdown facilitates the anti-tumor effect of CFG on HEY-T30 and SKOV3 cells. **(A, B)** Comparison of apoptotic cells in CFG-treated SKOV3 cells with/without PHF19 knockdown. A: Representative images. Scale bar: 200 μm. B: Quantitative analyses of early and late apoptotic cells. **(C)** Western blot to detect the expression of apoptosis-associated proteins BAD and BCL2 in CFG-treated SKOV3 cells with/without PHF19 knockdown. **(D, E)** Invasion and migration abilities were determined in CFG-treated SKOV3 cells with/without PHF19 knockdown. D: Representative image of cell migration and invasion. Scale bar: 100 μm. E: Quantitative results of migration and invasion assays. **(F)** The expression of the EMT markers, E-CADHERIN, N-CADHERIN and VIMENTIN, were determined in CFG-treated SKOV3 cells with/without PHF19 knockdown by Western blot analysis. **(G-I)** HEY-T30 and CFG-treated SKOV3 cells with PHF19 knockdown were subjected to a sphere formation assay. Scale bar: 200 μm. The number and size of tumor spheres were shown in H and I, respectively. The data are shown as average ± SD from three different experiments. ^*^, *P* < 0.05; ^**^, *P* < 0.01; ^***^, *P* < 0.001.

Figure S3 CFG does not affect the expression of miR-211 in ovarian cancer cells. **(A** and **B)** HEY-T30 **(A)** and SKOV3 **(B)** were treated with 0, 3 and 12 mg/ml of CFG for 24 h and the expression of miR-211 was determined by RT-qPCR.
