# Supplementary figures and images for: PHD Finger Protein 19 Enhances the Resistance of Ovarian Cancer Cells to Compound Fuling Granule by Protecting Cell Growth, Invasion, Migration, and Stemness
Source: Front Pharmacol. 2020 Feb 28;11:150. doi: 10.3389/fphar.2020.00150 (PMC7059104; doi:10.3389/fphar.2020.00150)

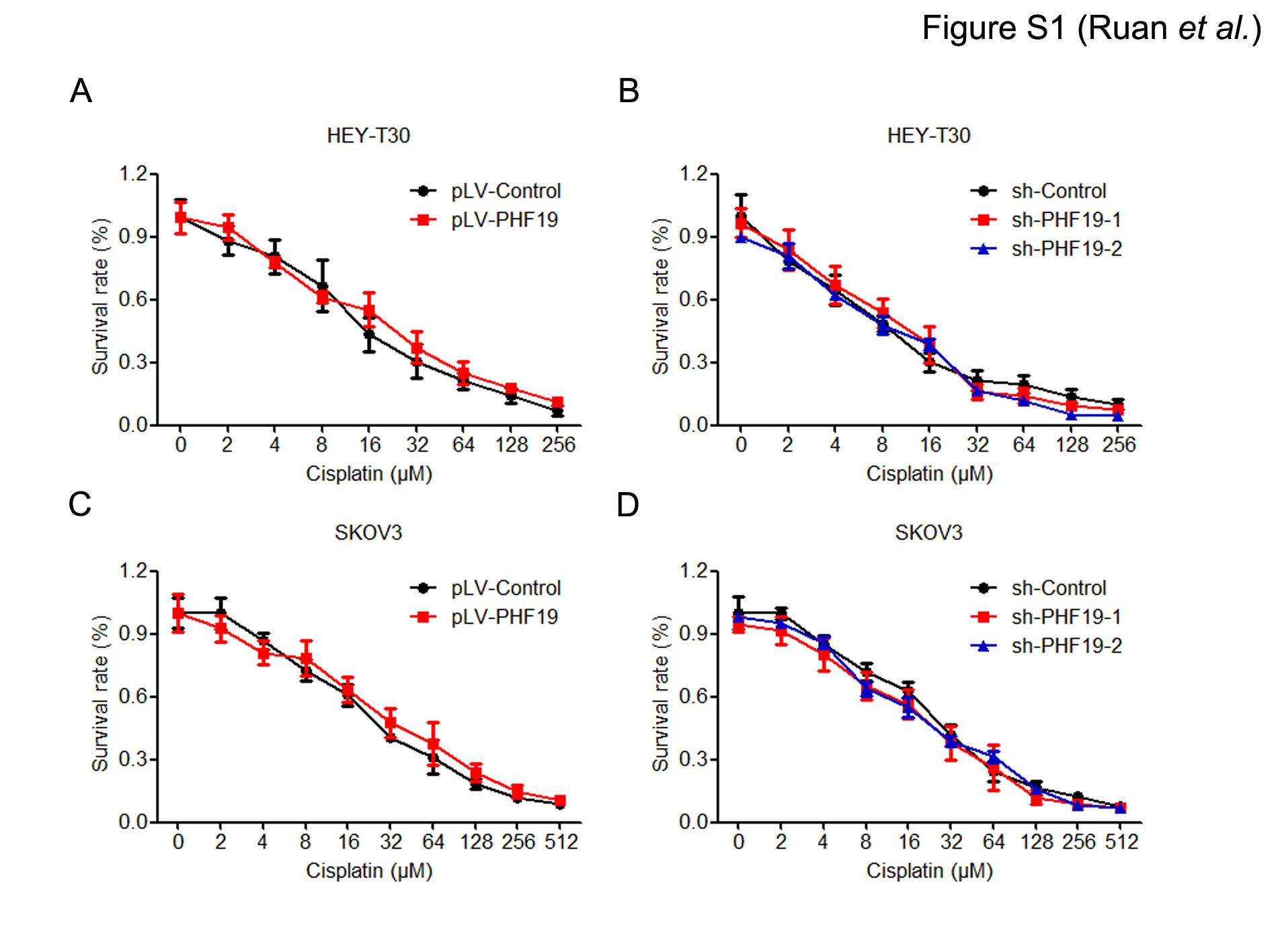

Supplement: Figure S1 — Changes of PHF19 expression cannot influence the anti-tumor effect of cisplatin in ovarian cancer HEY-T30 and SKOV3 cells. (A and C) HEY-T30 (A) and SKOV3 (C) cells with/without PHF19 overexpression were treated with different concentrations of cisplatin (0, 2, 4, 8, 16, 32, 64, 128 or 256 μM) and cell viability was detected by MTT assays. (B and D) Different concentrations of cisplatin (0, 2, 4, 8, 16, 32, 64, 128 or 256 μM) were added into the HEY-T30 (B) and SKOV3 (D) cancer cells with/without PHF19 knockdown and cell viability was assayed. The data are shown as average ± SD from three different experiments. [file Image_1.jpeg]

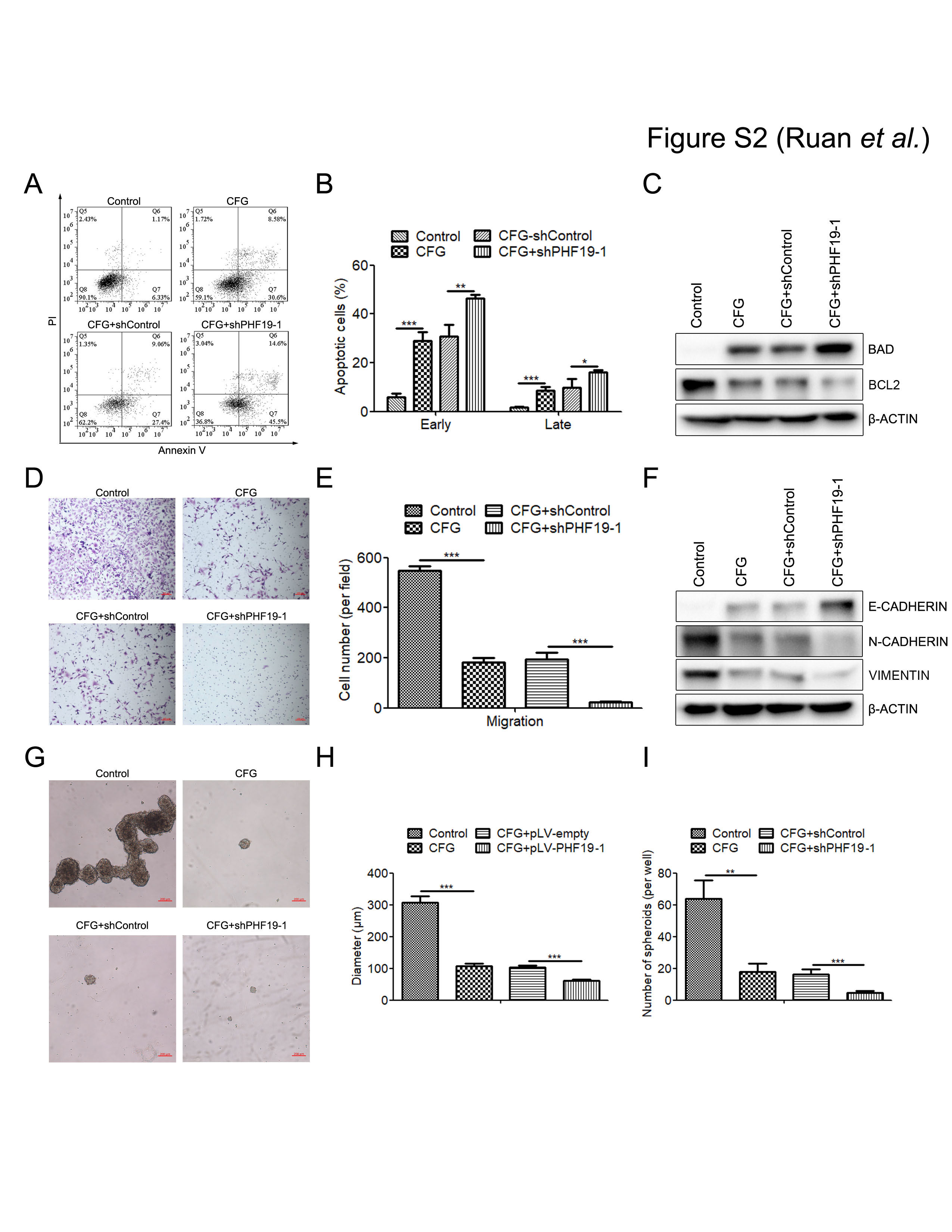

Supplement: Figure S2 — PHF19 knockdown facilitates the anti-tumor effect of CFG on HEY-T30 and SKOV3 cells. (A, B) Comparison of apoptotic cells in CFG-treated SKOV3 cells with/without PHF19 knockdown. A: Representative images. Scale bar: 200 μm. B: Quantitative analyses of early and late apoptotic cells. (C) Western blot to detect the expression of apoptosis-associated proteins BAD and BCL2 in CFG-treated SKOV3 cells with/without PHF19 knockdown. (D, E) Invasion and migration abilities were determined in CFG-treated SKOV3 cells with/without PHF19 knockdown. D: Representative image of cell migration and invasion. Scale bar: 100 μm. E: Quantitative results of migration and invasion assays. (F) The expression of the EMT markers, E-CADHERIN, N-CADHERIN and VIMENTIN, were determined in CFG-treated SKOV3 cells with/without PHF19 knockdown by Western blot analysis. (G-I) HEY-T30 and CFG-treated SKOV3 cells with PHF19 knockdown were subjected to a sphere formation assay. Scale bar: 200 μm. The number and size of tumor spheres were shown in H and I, respectively. The data are shown as average ± SD from three different experiments. *, P < 0.05; **, P < 0.01; ***, P < 0.001. [file Image_2.jpeg]

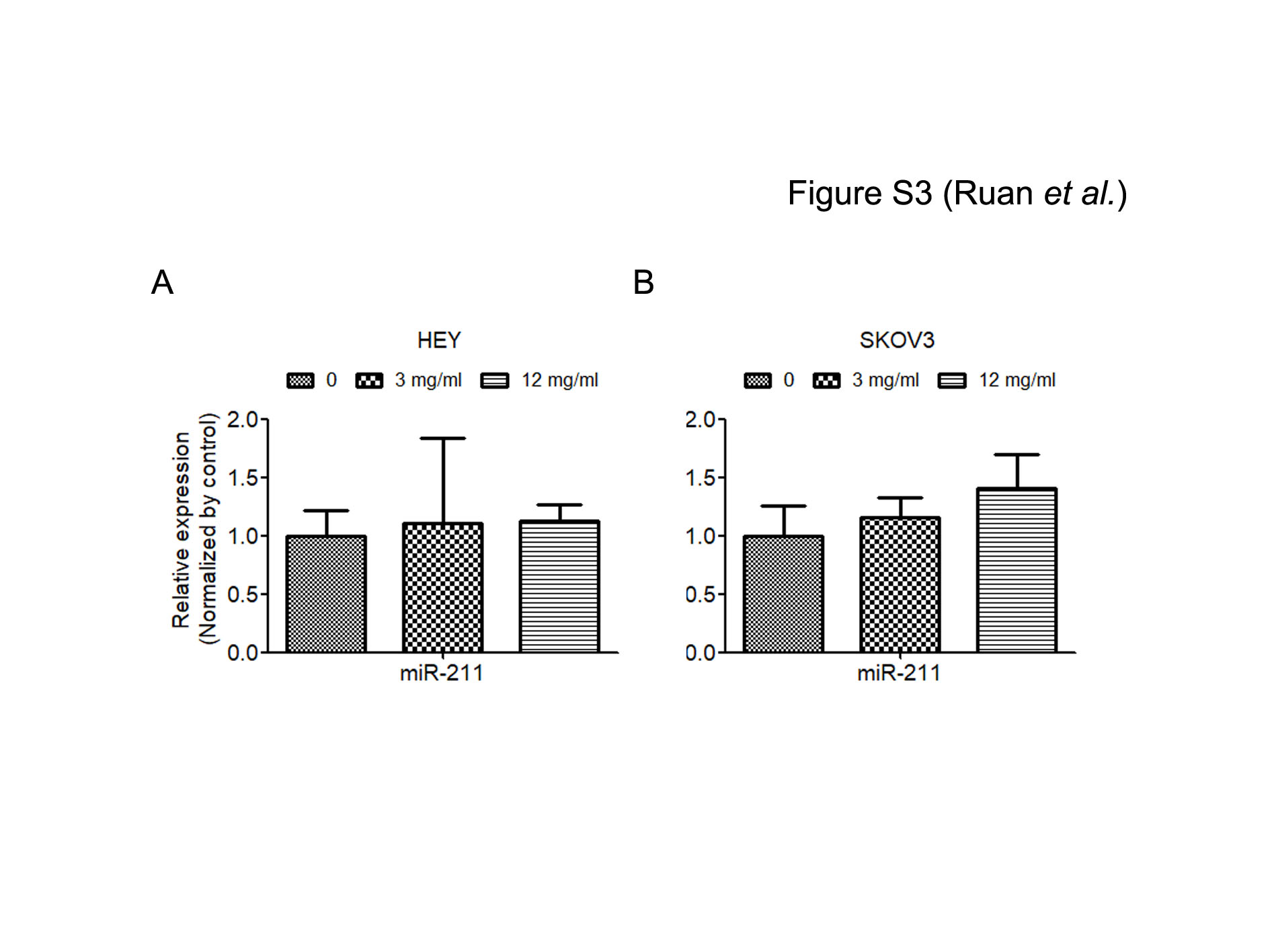

Supplement: Figure S3 — CFG does not affect the expression of miR-211 in ovarian cancer cells. (A and B) HEY-T30 (A) and SKOV3 (B) were treated with 0, 3 and 12 mg/ml of CFG for 24 h and the expression of miR-211 was determined by RT-qPCR. [file Image_3.jpeg]
